# Risk factors of musculoskeletal symptoms in university hospital nurses

**DOI:** 10.1186/s40557-014-0047-7

**Published:** 2014-11-04

**Authors:** Eunkwang Ryu, Byeongjin Ye, Youngil Yi, Jungwon Kim

**Affiliations:** Department of Occupational and Environmental Medicine, Kosin University College of Medicine, Gamcheonro 262, Seogu, Pusan, South Korea

**Keywords:** Nurses, Musculoskeletal, Symptom, Stress, Occupations

## Abstract

**Objectives:**

The purpose of this study was to investigate musculoskeletal symptom prevalence in university hospital nurses and explore the relation between musculoskeletal symptom prevalence and work related factors.

**Methods:**

A structured questionnaire was conducted with 620 nurses in a university hospital to look into the characters of responsibility and musculoskeletal symptom prevalence. The questionnaire respondents numbered 534, so the response rate was 86.1%. Among the respondents, three who gave insincere answers were excluded. The final study population was 531 respondents. ANSI Z-365 checklist was applied to look into ergonomic characteristics, and Korean Occupational Stress Scale Short Form was employed to measure job stress.

**Results:**

In the case of the whole body, symptom prevalence amounted to 70.8%. Regarding each body region, shoulder symptom prevalence accounted for the highest, or 44.8%, waist 40.7%, and neck 33.3% in order. According to multiple logistic regression analysis, in the case of the whole body, the group with a high ANSI checklist grade had odds ratio of 3.59 (95% CI 1.48 ~ 8.76), and the group with high job stress had 3.19 (95% CI 2.01 ~ 5.07).

**Conclusion:**

Regarding the occupational factors related to musculoskeletal symptoms of university hospital nurses, it was found that ANSI Z-365 checklist high risk group, total job tenure, department, shiftworks, and job stress had high relation with musculoskeletal symptoms. It is necessary to find an ergonomic solution and a stress reduction plan to prevent musculoskeletal disease.

## Introduction

Work-related musculoskeletal disease is one of occupational diseases recently increased by a new industrial structure and a changed social environment. It is a health problem caused by something related to repetitive motion, improper work posture, excessive use of force, the sharp side of the body contact, and vibration and temperature factor. It refers to a disease appearing in the nerves and muscles of the neck, the shoulder, the waist, and the upper and lower limbs, and in surrounding body tissues.

In the US, it was said that musculoskeletal disease is the main cause of the absence from work induced by occupational injuries and diseases [1]. According to the 2007–2009 occupational injuries and disease study in the UK, musculoskeletal disease accounted for 53% (the highest) of all diseases, and amounted to 27% (the second largest following mental diseases) in terms of the rate of absence from work. According to the 2010 2nd Korean working condition survey (the 2nd KWCS) with Korean workers, the absence from work induced by musculoskeletal disease accounted for 1.01% of total employees. It was the highest rate, around 53% among the rate of absence from work of total employees (1.8%) [2].

It is known that, among risk factors of work related musculoskeletal disease, ergonomic factors are important. According to National Institutes of Safety and Health (NIOSH), however, psychosocial factors, such as workload dissatisfaction, monotonous works, limited job autonomy, low job clarity, tedious work, and low social support, have something to do with a variety of work-related musculoskeletal disease [3].

The tasks of medical care workers in hospital vary. The risk factors of work-related disease are categorized into biological factors, physical factor, chemical factor, ergonomic factor, psychiatric factor, and psychological factors by task [4]. Of medical care workers, nurses, according to Occupational Safety and Health Agency (OSHA), are in the 10 major occupations which have the high risk of work-related musculoskeletal disease [5]. In Korea, they accounted for about 9%, or 106, of 1,181 health and human services workers who had been diagnosed with musculoskeletal disease from 2006 to 2009 [6].

The biggest cause of nurses’ musculoskeletal disease is the character of their works requiring excessive tension and concentration, such as always lifting heavy objects, standing work, and dealing with dangerous goods [7-10]. Another one is the character of their jobs requiring the postures damaging the waist and the body, such as bending, twisting hands, and dealing with patients [11,12]. Domestic nurses do repetitive jobs mainly using their upper limbs, like writing medical records and test records, and have the character of works requiring the frequent use of their lower body part in stations, which need improper and atypical postures, such as injection and medical care for patients. Also it is known that they receive psycho-social stress because of their job character of giving assistance for patient treatment, and experience severe conflict on account of the different work scope of occupations in hospital, where there are many different occupations [13].

The previous Korean studies of musculoskeletal disease focused on relevant factors. Most of them looked into only the relation between the disease and ergonomic factors or between the disease and psycho-social factors, or investigated work-related musculoskeletal disease of operation room nurses. There is few research on the risk factors of the disease by body region. The study on medical care workers in one university hospital published in this journal in 2012, which mentioned the issue partially, is the only case. In other words, there is no study on nurses related musculoskeletal disease. Therefore these author investigated musculoskeletal symptom prevalence in nurses in one university hospital, and looked into ergonomic factors, job stress, work-related factor, and risk factors by body region.

## Materials and methods

### Study population

This study conducted a structured questionnaire survey in a self-record type with 620 nurses in a university hospital located in Busan in 2011. The study subjects were those who heard the purpose of this study before their participation and agreed on the survey. The questionnaire respondents numbered 534, and the response rate was 86.1%. Among them, three respondents gave insincere answers. As a result, 531 respondents were selected as a study population.

### Study methods

The questionnaire includes personal history information, such as age, martial status, and house, and work-related factors, including occupation, department, position, total job tenure, works types, and character of works. Their age is classified into 20 something, 30 something, 40 something, and over 50 something. Their work department is classified into station, operation room, ICU, and other departments. Their work position is divided into temporary position and permanent position, and their work type into day works and shiftworks. Their position is classified into general nurse, responsibility nurse, and head nurse and over. Operation room includes operation room nurses. ICU room includes internal medicine part, surgery part, neurology part, and Intensive care units. Other departments include referral center, proper care center, clinical trials centre, outpatient, occupational health centers, infection control room, recovery room, artificial endoscopy laboratory, emergency department, supply chamber, cardiac laboratory, and nursery.

For the questionnaire about musculoskeletal symptom prevalence, this study employed the burden of musculoskeletal research work instructions of Korea occupation safety health agency (KOSHA code H-30-2008). With the tools, whether the study subjects experienced any pains on the neck, the shoulder, the arms/elbow, the hands/wrists/finger, the waist, knee, and the foot/ankle over the last one year was surveyed [14].

In reference to the data of National Institute of Occupational Safety & Health, the criterium of musculoskeletal symptom prevalence in the musculoskeletal questionnaire was defined as ‘the case where a symptom lasted more than one week or happened more than once every month over the last one year’.

For ergonomic factor, ANSI Z-365 Quick checklist of American National Standards Institute (ANSI) was employed [15]. The checklist is comprised of repeated exposure levels and repeat operation, the average weight at heavy work and exposure time, the push/pull operations when working strength, weight, and when moving heavy exposure, risk factors of posture (neck/ shoulder, hand stretch, elbow hand/wrist bending, twisting of the waist, knee: curled or use group work), using power tools, pressure body parts, a fixed work postures, work environment, keyboard work, and incentive/work speed control. Based on the sum of the points of each item, those with 0 ~ 9 points were categorized in low risk group; those with 10 ~ 15 points in moderate risk group; and those with more than 16 points in high risk group.

For job stress measurement, Korean Occupational Stress Scale-24 (KOSS-24) was employed. The KOSS-24 consists of such sub-elements as job demands, job autonomy, interpersonal conflict, job insecurity, inadequate compensation, organizational structure, and workplace culture. Job stress sub part and total job stress score were based on the 50th percentile reference value suggested by Chang et al. [16]. The job stress was measured in the categories of the group with low total job stress and the group with high stress.

This study was reviewed for private information protection and approved by the Institutional Review Board of the Kosin university hospital.

### Statistical analysis

Analysis of frequency was applied to the general characters, work-related characters, and psycho-social factor of all study subjects. And analysis of frequency was conducted on symptom prevalence in the categories of the whole body and body regions.

To investigate whether there are any differences in musculoskeletal symptom prevalence in the whole body and in body regions regarding general characters, work-related characters, and job stress, this study conducted Chi-square test and Fisher’s exact test.

Ergonomic characters, work related characters, and job stress were set to independent variables, and musculoskeletal symptom prevalence and positive symptom according to the whole body and each body region were set to dependent variables. With these variables, linear logistic regression analysis was performed. With significant independent variables, age was adjusted first and then multiple logistic regression analysis was conducted. The assumptions of multiple regression analysis were verified. As a result, tolerance was less than 1.0; and variation inflation factor and VIF didn't exceed 10. Confidence interval was set to 95%, and significance level to less than 0.05. For statistical analysis, 18.0 SPSS 18.0 Statistics program was used.

## Results

### Characters of study population characters

Regarding the age distribution of study subjects, those in their 20s accounted for the highest or 56.1%, followed by those in their 30s, 40s, and 50s in order. 68.0% of the study subjects were ‘unmarried’. Regarding house-working hours, those with ‘less than 3’ house-working hours accounted for 75.7%, followed by those with ‘3 to 5 hours’ and those with ‘more than 6 hours’ in order. Regarding department, those working at ‘station’ accounted for 65.9%, those working at ‘operation room’ 6.6%, those working at ‘ICU’ 13.0%, and those working at ‘others’ 14.5%. With regard to total job tenure, those with ‘less than 5 years’, those with ‘5 to 10 years’, and those with ‘more than 10 years accounted for 50.3% (the largest), 24.7%, and 25.0%, respectively. Regarding position, ‘general nurses’ accounted for the highest percentage. Regarding work character, those with ‘shiftworks’ accounted for the highest, or 77.6 %. With regard to ergonomic characteristics based on ANSI Z-365 evaluation classification, those with ‘low risk grade’, those with ‘moderate risk grade’, and those ‘high-risk garde’ accounted for 71.4% (the largest), 17.1%, and 11.5%, respectively (Table 1). Regarding total job stress, they were in 25th to 50th percentile.

**Table 1:** **Characteristics of study population**

**Variables**		**Number of subjects(%)**	
		**N**	**%**
Age(year)	20 ~ 29	298	56.1
	30 ~ 39	144	27.1
	40 ~ 49	74	13.9
	≧50	15	2.8
Martial status	Unmarried	361	68.0
	Married	168	31.6
	other	2	0.4
House work hour per day	0 ~ 2	402	75.7
	3 ~ 5	91	17.1
	≧6	38	7.2
Department	Ward	350	65.9
	Operation room	35	6.6
	Intensive care unit	69	13.0
	Other	77	14.5
Shiftwork	No	121	22.4
	Yes	410	77.6
Tenure(years)	0 ~ 4	267	50.3
	5 ~ 10	131	24.7
	>10	133	25.0
Position	General duty nurse	428	80.6
	Responsibility nurse	56	10.5
	Head nurse	47	8.9
Job satisfaction	Very satisfied	37	7.0
	Satisfied	343	64.6
	Unsatisfied	143	26.9
	Very unsatisfied	8	1.5
ANSI-Z 365 checklist	Low grade	379	71.4
	Moderate grade	91	17.1
	High grade	61	11.5

### Musculoskeletal symptom prevalence of the study population

Based on the criterium that symptom prevalence is defined as ‘the case where a symptom lasted more than one week or happened more than once every month over the last one year’, in the case of the whole body, symptom prevalence amounted to 70.8%, and in the case of each body region, shoulder symptom prevalence amounted to the highest, or 44.8%; waist symptom prevalence 40.7%; neck symptom prevalence 33.3%; and knee symptom prevalence 30.1%; hands/wrists/finger symptom prevalence 17.5%; foot/ankle symptom prevalence 14.9%; and arm/elbow symptom prevalence 5.5%. In the case of age, those in their 30s had the highest percentage of symptom prevalence, and those in their 50s the lowest. Unmarried study subjects had a higher percentage of symptom prevalence than married ones. Regarding house-working hours per day, those with more than six hours had the highest percentage of symptom prevalence. With regard to department, operation room nurses had the highest (74.3%) of symptom prevalence. Regarding total job tenure, those with less than four years had the highest percentage (71.5%) of symptom prevalence. Regarding position, general nurses had the highest (71.7%) of symptom prevalence. According to ANSI Z-365 evaluation table, those with high risk grade had 가 symptom prevalence 90.2% (the highest) of symptom prevalence. The group with high total job stress had a higher percentage of symptom prevalence than the group with low job stress (Table 2).

**Table 2:** **Whole body musculoskeletal symptoms of study population**

		**Symptoms**				
**Variables**		**Negative**		**Positive**		**p-value**
		**N**	**%**	**N**	**%**	
Age	20 ~ 29	86	28.9	212	71.1	0.225
	30 ~ 39	40	27.8	104	72.2	
	40 ~ 49	21	28.4	53	71.6	
	≧50	8	53.3	7	46.7	
Martial status	Unmarried	101	28.0	260	72.0	0.459
	Married	54	32.1	114	67.9	
Shiftwork	No	42	32.1	89	67.9	0.036
	Yes	113	28.3	287	71.8	
House work hour per day	0 ~ 2	117	29.1	285	70.9	0.994
	3 ~ 5	27	29.7	64	70.3	
	≧6	11	28.9	27	71.1	
Department	Ward	103	29.4	247	70.6	0.962
	Operation room	9	25.7	26	74.3	
	Intensive care unit	21	30.4	48	69.6	
	Others	22	28.6	55	71.4	
Tenure	0 ~ 4	70	28.5	176	71.5	0.089
	5 ~ 10	41	28.9	101	71.1	
	>10	44	30.8	99	69.2	
Position	General duty nurse	121	28.3	307	71.7	0.375
	Responsibility nurse	16	28.6	40	71.4	
	Head nurse	18	38.3	29	61.7	
ANSI Z-365 checklist	Low grade	127	33.5	252	66.5	0.000
	Moderate grade	22	24.2	69	75.8	
	High grade	6	9.8	55	90.2	
Total job stress	Low	125	37.9	205	62.1	0.000
	High	30	14.9	171	85.1	

### The relation between work related characters and musculoskeletal symptom prevalence

According to linear logistic regression analysis, ANSI checklist grade, department, total job tenure, shiftworks, and total job stress had relation. According to multiple logistic regression analysis, in the case of the whole body, the group with high ANSI checklist grade had odds ratio of 3.59 (95% CI 1.48 ~ 8.76), and those with job stress had odds ratio of 3.19(95% CI 2.01 ~ 5.07). In the case of the neck, those with 5 to 10 work years had odds ratio of 1.72(95% CI 1.05 ~ 2.85). In the case of the shoulder, those with more than 10 work years had odds ratio of 1.77(95% CI 1.09 ~ 2.88). In the cases of the arm/elbow, the hands/wrists/finger, and the knee, operation room nurses had odds ratio of 4.10(95% CI 1.21 ~ 13.90), of 2.66(95% CI 1.10 ~ 4.90), and of 3.87(95% CI 1.67 ~ 8.81), respectively. In the case of the foot/ankle, those with shiftwork had odds ratio of 4.28(95% CI 1.70 ~ 4.86). Those with high job stress had odds ratio of 3.19(95% CI 2.01 ~ 5.07) in the whole body, of 1.93(95% CI 1.32 ~ 2.83) in the neck, 1.98(95% CI 1.37 ~ 2.85) in the shoulder, of 3.30(95% CI 1.46 ~ 7.50) in the arm/elbow, of 2.66(95% CI 1.63 ~ 4.32) in the hands/wrists/fingers, of 2.37(95% CI 1.64 ~ 3.43) in the waist, and of 2.00(95% CI 1.35 ~ 2.96) in the knee (Table 3). Other body part had odds ratio of 2.33(95% CI 1.11 ~ 4.91) in the hand/wrist/finger, of 1.99(95% CI 1.01 ~ 3.94) in the waist, of 5.22(95% CI 2.02 ~ 13.52) in the foot/ankle.

**Table 3:** **Odds ratios of work-related factors for musculoskeletal symptoms by multivariate analysis**

		**Unadjusted OR**		**Adjusted OR** ^*^	
**Variables**		**OR**	**95% CI**	**OR**	**95% CI**
Whole body					
ANSI-Z 365 checklist	Low	1.00		1.00	
	Moderate	1.58	0.94-2.67	1.28	0.74-2.27
	High	4.62	1.94-11.01	3.59	1.48-8.76
Tenure(year)	0 ~ 4				
	5 ~ 10	0.98	0.62-1.55	0.80	0.47-1.37
	>10	0.89	0.57-1.40	0.86	0.35-2.11
Department	Ward				
	Operation room	1.20	0.55-2.67	1.08	0.43-2.77
	ICU^†^	0.95	0.54-1.68	0.92	0.50-1.66
	Other	1.04	0.60-1.80	1.22	0.60-2.49
Shiftwork	No				
	Yes	1.20	0.78-1.84	1.30	0.68-2.50
Total job stress	Low				
	High	3.48	2.22-5.44	3.19	2.01-5.07
Neck					
Tenure(year)	0 ~ 4				
	5 ~ 10	1.87	1.20-2.90	1.72	1.05-2.85
	>10	1.74	1.12-2.70	1.94	0.85-4.41
Department	Ward				
	Operation room	1.84	0.91-3.71	2.06	0.91-4.63
	ICU^†^	0.77	0.43-1.38	0.80	0.44-1.46
	Other	1.63	0.99-2.71	1.80	0.93-3.50
Shiftwork	No				
	Yes	0.86	0.85-1.30	1.50	0.81-2.79
Total job stress	Low			1	
	High	1.96	1.36-2.84	1.93	1.32-2.83
Shoulder					
Tenure(year)	0 ~ 4				
	5 ~ 10	1.30	0.85-1.97	1.20	0.78-1.84
	>10	1.74	1.15-2.64	1.77	1.09-2.88
Department	Ward				
	Operation room	1.51	0.75-3.03	1.40	0.64-3.03
	ICU^†^	0.72	0.42-1.23	0.77	0.46-1.33
	Other	1.38	0.84-2.26	1.17	0.62-2.19
Shiftwork	No				
	Yes	0.74	0.50-1.10	1.06	0.61-1.86
Total job stress	Low				
	High	1.91	1.34-2.72	1.98	1.37-2.85
Arm/elbow					
Tenure(year)	0 ~ 4				
	5 ~ 10	1.38	0.78-2.43	0.90	0.46-1.76
	>10	2.09	1.23-3.55	0.93	0.34-2.55
Department	Ward				
	Operation room	3.96	1.42-11.06	4.10	1.21-13.90
	ICU^†^	0.61	0.14-2.76	0.81	0.17-3.75
	Other	1.02	0.23-4.68	1.52	0.46-4.92
Shiftwork	No				
	Yes	0.44	0.20-0.95	0.96	0.33-2.76
Total job stress	Low				
	High	2.85	1.32-6.17	3.30	1.46-7.50
Hand/wrist/finger					
Tenure(year)	0 ~ 4				
	5 ~ 10	1.38	0.78-2.43	0.90	0.45-1.76
	>10	2.10	1.23-3.56	0.93	0.34-2.56
Department	Ward				
	Operation room	3.06	1.43-6.53	2.66	1.10-4.90
	ICU^†^	0.49	0.18-1.20	0.46	0.17-1.21
	Other	2.81	1.61-4.94	2.33	1.11-4.91
	High				
Shiftwork	No				
	Yes	0.44	0.27-0.71	0.92	0.46-1.86
Total job stress	Low				
	High	2.33	1.49-3.67	2.66	1.63-4.32
Waist					
Tenure(year)	0 ~ 4				
	5 ~ 10	1.01	0.66-1.52	0.86	0.53-1.40
	>10	0.64	0.41-0.98	0.83	0.37-1.85
Department	Ward				
	Operation room	1.05	0.52-2.12	1.41	0.61-3.24
	ICU^†^	0.70	0.41-1.20	0.66	0.38-1.17
	Other	1.00	0.60-1.64	1.99	1.01-3.94
Shiftwork	No				
	Yes	1.56	1.03-2.36	1.83	0.97-3.43
Total job stress	Low				
	High	2.47	1.72-3.54	2.37	1.64-3.43
Knee					
Tenure(year)	0 ~ 4				
	5 ~ 10	1.03	0.66-1.62	0.77	0.45-1.30
	>10	1.05	0.67-1.65	0.74	0.32-1.73
Department	Ward				
	Operation room	3.91	1.91-8.00	3.87	1.67-8.81
	ICU^†^	0.79	0.43-1.44	0.78	0.42-1.44
	Other	1.33	0.79-2.25	1.45	0.73-2.88
Shiftwork	No				
	Yes	0.74	0.48-1.12	1.06	0.57-1.99
Total job stress	Low				
	High	1.99	1.36-2.90	2.00	1.35-2.96
Foot/ankle					
Tenure(year)	0 ~ 4				
	5 ~ 10	0.70	0.40-1.28	0.49	0.24-1.00
	>10	0.74	0.41-1.33	0.61	0.20-1.83
Department	Ward				
	Operation room	1.21	0.48-3.06	2.34	0.80-6.90
	ICU^†^	0.56	0.23-1.36	0.53	0.22-13.5
	Other	1.53	0.83-2.87	5.22	2.02-13.52
Shiftwork	No				
	Yes	1.78	1.23-3.37	4.28	1.70-4.86
Total job stress	Low				
	High	2.84	1.74-4.64	1.16	0.30-4.41

^*^odds ratio adjusted for age, ANSI Z-365 checklist, tenure, department, shiftwork, total job stress.

^†^Intensive care unit.

## Discussion

This study tried to investigate the relation with ergonomic risk factors, job stress, and work-related factor of nurses according to body regions. In the case of the whole body, ANSI checklist grade had relation. In the case of each body region, work tenure, department, shiftworks, and job stress had relation.

According to the study on workers’ musculoskeletal symptom prevalence based on the same criterium as this study, the musculoskeletal symptom prevalence of electronic component assembly female workers was 80.9%; that of food manufacturing workers 64.2%, and that of textile sewing up women workers is. The results were similar to the prevalence 70.8% in this study. According to the study of male and female workers in the main shipbuilding industry by bakjeongseon, the musculoskeletal symptom prevalence of male manufacturing workers was 64.3%, and that of female ones was 69.2%. The results were not higher than the musculoskeletal symptom prevalence of university hospital nurses in this study.

This study was compared with previous studies on hospital nurses. In the study by Woh [17] et al., the whole body symptom prevalence was 66.8%; in the study by Park [18] et al., it was 79.0%. In the study by Woh et al., symptom prevalence was high in the order of the knee, the shoulder, and the waist, whereas in the study by Park et al., it was high in the order of the shoulder, the knee, the foot/ankle, and the waist. In this study, symptom prevalence was high in the order of the shoulder, the waist, the neck, and the knee. Depending on studies, there were differences in the order of body regions and symptom prevalence. It is considered that the causes are different working conditions and different labor grades. Another critical cause is the difference in the definition of symptom prevalence and diagnosis method depending on surveyors. Nevertheless, the result that each study showed symptom prevalence in the almost same body regions indicates that the work types of nurses are reflected well.

The relation between the body symptom prevalence and wok-related factor relation was analyzed. As a result, in the case of the whole body, as an ANSI checklist grade went up, odds ratio of symptom prevalence significantly increased to 3.59(95% CI 1.48 ~ 8.76). A previous study observed neck symptom according to the total score, and revealed that high risk group increased relative risk of musculoskeletal symptom more than low risk group [19]. According to the study of Choi et al. on the medical staff of a university hospital, the higher male and female medical staff had an ANSI checklist grade, the higher their relative risk became [20]. The result is presumed to be related to the characteristics of hospital work described earlier, and supports the conclusion that ergonomic work environment improvement is necessary.

In the cases of the neck and the shoulder, a rise in total job tenure had significant relation with odds ratio of symptom prevalence. According to Kurumatani, the longer the tenure was, the higher the perception symptom prevalence for body regions, such as the shoulder, the neck, and the arms became [21]. The study by Park revealed that the group of workers with more than five work years had statistically significant higher musculoskeletal symptom prevalence than the group with less than five work years [22]. The pains on the neck and the shoulder, and lethargy of the arms had relation with tenure, and the pain on the waist had significant relation with a rise in tenure [22]. Kourinka and Forcier proved that a level of exposure per day or in lifetime increased the strength of the relation between exposure and work-related musculoskeletal disease [23]. This study also revealed that in the cases of the neck, the shoulder, and the arms/elbows, tenure had significant relation with symptom prevalence.

In the cases of the arms/elbows, the hands/wrists, and the knees, odds ratio of symptom prevalence in operation room nurses was 4.10(95% CI 1.21 ~ 13.90), 2.66(95% CI 1.10 ~ 4.90), and 3.87(95% CI 1.67 ~ 8.81), respectively. Operation room nurses need to make a quick and accurate judgment, repeatedly use one arm, lift or move a heavy object, and take a fixed posture and tension for a long time in the process of surgical operation. Therefore, they have a lower level of health conditions than other workers [24]. The scrub work of operation room nurses is to prepare operation tools and hand over the prepared tools to operating surgeons. It requires a fixed standing posture for a long time and a posture of keeping a certain distance. As a result, it causes unnatural postures. It was reported that the nurses take a fixed posture for a long time and bend their neck in operation, use repeatedly their wrists and fingers to access operation tools, and intermittently give excessive power (to use mosquito, kelly, etc.) [25,26]. In other studies, REBA evaluation analysis showed that among their works, preoperative disinfection of goods and utensils ready, push disinfection article ,‘ related to supply of goods surgery ’,‘ machinery, equipment delivered to the surgical team during surgery ”, and ‘ preparing surgical equipment, connect and disconnect operation ’had a high level of risk [27]. Since operation room nurses continuously work for a long time, their body fatigue can be accumulated. Therefore, it is necessary to provide appropriate shiftworks for them, give work-based stretching education to them before and after operation and during rest time, and come up with an ergonomic improvement plan to reduce the burden of a posture.

In the case of the foot/ankle, odds ratio, when shift-works were provided, was 4.28 (95% CI 1.70 ~ 4.86), significantly high. According to the study on Iranian nurses, symptom prevalence in the ankles and other body regions than non-shift workers [28]. It was reported that the rise of musculoskeletal disease of medical care workers had relation with shiftworks, and had relations with an increase in work hours and a decrease in rest time, caused by shiftworks [29]. The result shows that it is necessary to manage the works of shiftworkers for their health. In other words, it is necessary to reduce shiftworks, work the way forward, and provide enough rest time to them after work hours.

Other departments had significantly higher odds ratio of symptom prevalence in the hands/wrists/fingers, waist and the foot/ankle than general station. According to Lagerström et al., musculoskeletal symptom prevalence was different depending on station [30]. In this study, other departments mean small-sized departments excluding general station, operation room, and ICU, and they have different types of jobs and various characteristics. Therefore, it is difficult to make an analysis according to the hands/wrists/fingers, the ankles, and the waist. If more study objects are collected, it will be possible to perform an additional study and make a discussion.

Regarding job stress, in most body regions except for the foot/ankle, such as the neck, the shoulder, the arm/elbow, the hands/wrists/fingers, the back/waist, and the knees, the group with high job stress had significantly high odds ratio. According to said Bongers et al., the mechanism of musculoskeletal disease is attributable to the continuance of physical working factors induced musculoskeletal inflammation by stress, or the tension of muscles or muscular reduction of the pain threshold caused by job stress [31]. The domestic study by Woh et al. showed that job stress statistically significantly affected the shoulder [32]. The study by Kim et al. revealed that nurses’ job stress statistically significantly influenced the shoulder, the arms, the hands, the waist, and other body regions [33]. The study by Park et al. reported that job stress has greatly significant relation with musculoskeletal symptom in the elbows and the knees among body regions. It is considered that it will be necessary to survey the works which can trigger a lot of job stress, and suggest a solution tailored to each work.

To manage nurses’ musculoskeletal disease, it will be essential to analyze approved industrial accident data, evaluate risk factors exposure, additionally survey and study work environment improvement cases, and make nurses who have musculoskeletal symptom and managers involved in finding a solution. When harmful factors and risk factors are analyzed, it is necessary to manage and improve musculoskeletal disease and job stress in the participation of those concerned [6].

This study has the following limitations:

It used the data of one university hospital so that it is hard to generalize the study results. Therefore, it will be necessary to survey various medical care centers and reconfirm the results of this study. The self-report typed questionnaire was applied to look into musculoskeletal symptom. Therefore, there is the possibility that individuals’ subjective judgment would be involved, and there would be difference with clinical diagnosis. Since this is cross-sectional study, there are limitations in investigating the accurate causal relation between musculoskeletal symptom and relevant factors [34]. Therefore, it will be necessary to conduct an additional study to overcome the limitations.

Occupational musculoskeletal disease breaks out in a complex way by various factors. So it is very important to find and prevent risk factors early. The occurrence frequency of musculoskeletal symptom, and the relation between symptom prevalence and risk factors according to body regions, mentioned in this study, will be conducive to understanding the musculoskeletal disease and job stress of medical care nurses, and will help employers, workers, health and safety managers, and policy chairs to suggest relevant policies and musculoskeletal disease prevention projects.

## Conclusion

Regarding the occupational factors related to musculoskeletal symptoms of university hospital nurses, it was found that ANSI Z-365 checklist high risk group, total job tenure, department, shiftworks, and job stress had high relation with musculoskeletal symptoms. It is necessary to find an ergonomic solution and a stress reduction plan to prevent musculoskeletal disease.
